# Policy Adoption and the Implementation Woes of the Intersectoral First 1000 Days of Childhood Initiative, In the Western Cape Province of South Africa

**DOI:** 10.34172/ijhpm.2020.173

**Published:** 2020-09-17

**Authors:** Ida Okeyo, Uta Lehmann, Helen Schneider

**Affiliations:** ^1^Department of Community and Health Sciences, School of Public Health, University of the Western Cape, Cape Town, South Africa.; ^2^UWC/SAMRC Health Services to Systems Research Unit, School of Public Health, University of the Western Cape, Cape Town, South Africa.

**Keywords:** Policy Analysis, Interests, Intersectoral Initiatives, South Africa, Implementation, Actors

## Abstract

**Background:** There is a growing interest in implementing intersectoral approaches to address social determinants especially within the Sustainable Development Goals (SDGs) era. However, there is limited research that uses policy analysis approaches to understand the barriers to adoption and implementation of intersectoral approaches. In this paper we apply a policy analysis lens in examining implementation of the first thousand days (FTD) of childhood initiative in the Western Cape province of South Africa. This initiative aims to improve child outcomes through a holistic intersectoral approach, referred to as nurturing care.

**Methods:** The case of the FTD initiative was constructed through a triangulated analysis of document reviews (34), in depth interviews (22) and observations. The analysis drew on Hall’s ‘ideas, interests and institutions’ framework to understand the shift from political agendas to the implementation of the FTD.

**Results:** In the Western Cape province, the FTD agenda setting process was catalysed by the increasing global evidence on the life-long impacts of brain development during the early childhood years. This created a window of opportunity for active lobbying by policy entrepreneurs and a favourable provincial context for a holistic focus on children. However, during implementation, the intersectoral goal of the FTD got lost, with limited bureaucratic support from service-delivery actors and minimal cross-sector involvement. Challenges facing the health sector, such as overburdened facilities, competing policies and the limited consideration of implementation realities (such as health providers’ capacity), were perceived by implementing actors as the key constraints to intersectoral action. As a result, FTD actors, whose decision-making power largely resided in health services, reformulated FTD as a traditional maternal-child health mandate. Ambiguity and contestation between key actors regarding FTD interventions contributed to this narrowing of focus.

**Conclusion:** This study highlights conditions that should be considered for the effective implementation of intersectoral action - including engaging cross-sector players in agenda setting processes and creating spaces that allow the consideration of actors’ interests especially those at service-delivery level. Networks that prioritise relationship building and trust can be valuable in allowing the emergence of common goals that further embrace collective interests.

## Background

The challenge of adopting and implementing policies once issues get onto government policy agendas (agenda setting) is a phenomenon that has long been of interest to health policy analysists.^1,2^ Despite the range of existing theories and studies on processes of agenda setting,^3-5^ adoption and implementation,^2,6^ there is a wide recognition of the limited empirical research of this nature in low- and middle-income contexts.^7,8^ Policy adoption, here used interchangeably with formulation, refers to the stage of the policy-making where policy alternatives are considered, including the allocation of responsibilities and resources.^8^ Implementation involves the translation of evidence into effective policy, which is both a common policy challenge^6,9-11^ and a particular challenge for intersectoral policies addressing the social determinants of health (SDH).^12,13^

The range of obstacles to effective implementation of SDH policies include the multifaceted nature of socio-economic factors that rarely offer clear policy solutions and often result in the lack of consensus on appropriate interventions.^14^ In addition, the use of a long-term life course approach in addressing SDH often does not align with electoral cycles and timelines of policy-makers.^14^ Efforts to intervene are also hindered by the dominance of biomedical perspectives that have established patterns of interests and power that determine the allocation of resources.^12^ The logistics surrounding intersectoral action for health, where the health sector works with other sectors, further complicates the above processes largely due to the historical and current organisation of bureaucracies as silos.^14-16^ Experiences of working intersectorally have also highlighted problems of lack of ownership or territorial ownership over particular policy issues,^13,17^ lack of accountability,^15^ limited resources and time to enable collaboration^11^ and the challenges of measuring effectiveness and impact.^19^ Even when legislative processes demand intersectoral working, there is rarely enforcement and dedicated resources towards integrated policy development.^15^

There is, then, increasing recognition of the need to understand the barriers to policy adoption and implementation of SDH policies by using policy analysis methodologies and theory.^10,12,20^ Such theory is relevant as it examines the influence of socio-economic and political contexts, and the complex interactions between actors and the content of the policy, including the influence of ideas.^21^

This paper aims to contribute to understanding intersectoral policy adoption and implementation by examining the policy processes associated with the first 1000 days (FTD) of childhood initiative, an intersectoral initiative aimed at addressing social determinants of child health, in the Western Cape province of South Africa. Focusing on the FTD experience, our research question examines why intersectoral approaches are not adopted and implemented as desired, despite what appears to be successful agenda setting. We focus on the stages of policy adoption and early implementation and aim to provide insights into what conditions may enable or inhibit the implementation of intersectoral approaches.

There have been limited empirical studies that have applied policy analysis perspectives in examining SDH policy experiences.^20^ Our work mirrors studies that have documented the challenges facing commitment to nutrition agendas during processes from agenda setting to implementation.^10^ Others have applied policy analysis methods to understand the implementation of the Health in All Policies (HiAP) approach, including factors that prompt agenda setting for HiAP,^22^ the influence of ideas, institutions and actors on the commitment of other sectors to HiAP,^23^ and what hinders formulation and implementation of HiAP initiatives.^9^ These studies also reveal that the reality of policy-making for wicked problems involves a series of continuously interacting factors that policy-makers often need to juggle at the same time such as defining policy problems, negotiating policy interventions, ascertaining trade-of costs and benefits, evaluating policies, among others.^10,16,22,23^

This work is relevant due to the renewed interest in intersectoral collaboration within the Sustainable Development Goals (SDGs) era, as an essential approach to tackle health inequalities.^24^ An intersectoral approach is also crucial for early childhood development (ECD) where evidence shows that integrated health, nutrition and stimulation interventions promote positive outcomes from early years to adulthood.^25^ The commission on the SDH and accompanying case studies highlighted the injustice of avoidable health inequalities and proposed that addressing ECD can have a significant impact on the entire life course.^26,27^ The FTD period, between birth and two years, has received increasing attention due to the development that occurs in all domains (sensory, motor and cognitive) in this period. The FTD therefore presents a window of opportunity for multisector ECD-related interventions that ensure a nurturing environment for adequate child development.^25,28^

The Nurturing Care Framework^29^ and the Global Strategy for Women’s, Children’s and Adolescents Health^30^ have set the roadmap for creating an enabling environment that supports child well-being across sectors. Further studies focused on effective ECD interventions in low- and middle-income contexts have shown the value of programs that address parenting, early education, and nutrition support on ECD.^31-33^ The Nurturing Care Framework for example, presents a set of interrelated components targeting stimulation, responsiveness and safety, enabled by social and political contexts that can provide an adequate environment for developmental progression.^29^

In South Africa, ECD and the FTD period are prioritised in the National Development Plan^34^ and the National Integrated Early Childhood Development Policy,^28^ which have highlighted action in ECD as crucial in ensuring national development and growth. Actors within the Western Cape province have recognised the significance of the FTD period in ensuring wellness and enabling children to thrive and reach their full potential.^35^ Although noted as performing better than other provinces in South Africa in terms of child health indicators, 37% of children in the Western Cape live in poor households (earning a monthly income below US$81.01) and 11% live in households where hunger is reported, making them vulnerable to poor developmental outcomes.^36^ The province has the highest rates of drug-related crime in the country with high levels of alcohol and substance abuse as the main contributing factors to child abuse.^37,38^ As a response to the growing number of at-risk children and these major social challenges, the province launched the FTD Initiative in 2016 to improve outcomes for children in terms of nutrition, health, education, caregiver support and protection and safety.^35^

## Methods

The study adopted a qualitative research methodology and a case study approach^39,40^ to analyse the adoption and implementation processes of the FTD initiative in the Western Cape province. Data were collected between May 2018 and August 2019 through document reviews, in-depth interviews and observations. Table 1 provides an overview of the data collection activities and of the study participants.

**Table 1:** Data Collection Processes

**Data Collection Processes**	**N**	**Examples**
**Observed processes**		**(Events and policy communities)**
FTD-related workshops	3	CBS workshop (*September 2017*) Drakenstein Parent Support Package Site Visit (*August 2018*) Nurturing Care Framework Workshop (*August 2018*)
Policy communities associated with the FTD	3	PICH working group consists of members from the Departments of Health, Social Development and Education, academics and NGOs. Provides platform for sharing insights and fostering collaboration between various partners *(Observed seven meetings between 2017 and 2019)* CBS group that meets bimonthly to discuss possible ways of organising CBS for the FTD and engages with district and CBS as well as academics *(Observed four meetings between 2017 and 2018)* FTD executive committee responsible for organising formulation processes of the FTD. They consist of deputy directors of nutrition, women’s and children’s health, a senior clinician and a member of the communications directorate of the health sector (*Observed one meeting of the core FTD committee in 2018*)
**Key informant interviews**		
Government sector (Health)	10	Provincial policy-makers, members of the FTD executive committee, district and sub district actors
Government sector (Social Development)	3	Representatives of the Department of ECD
NGOs and Civil Society	5	Largely part of the PICH group
Academics	4	Associated with the PICH and CBS groups
Total	22	

Abbreviations: FTD, first thousand days; PICH, Parent, Infant and Child Health; NGOs, non-governmental organisations; CBS, community-based services; ECD, early childhood development.

Observation methods are an established qualitative method of inquiry rooted in ethnographic research that help the researcher understand actor behaviour and processes occurring in context.^39^ Direct observations of relevant meetings were conducted to learn which stakeholders were involved, their levels of engagement and influence and how interventions were prioritised. The researcher attended and observed meetings of two working groups (the Parent, Infant and Child Health [PICH] working group and the community-based services group) and three FTD-related workshops (Table 1).

Although observations were useful to establish familiarity with key informants and to access key documents, we acknowledge the limitations of this method in studying processes of policy-making, especially as the emergence of decisions can be hard to identify within widespread networks of actors.^41^ Observational data is also subject to researcher bias which we accounted for by triangulating field notes with interviews and documentary evidence.

Documents reviewedincluded minutes of the meetings and workshops, official policy documents from the provincial website, research newsletters covering the FTD and annual reports from all relevant departments. National level policies that focused on the FTD, including maternal and child health policies and relevant scientific literature, were also included. Additional literature was sourced through hand searches of references in these documents. A total of 34 documents were analysed and data extracted provided information on administrative procedures, proposed interventions, actor involvement and collaborative engagements.

We aimed to interview key informants that were involved in the adoption and early implementation processes of the FTD. An initial list of key informants was provided by one of the members of the Community-Based Services (CBS) working group while other informants were identified through snowballing. Interviews continued until saturation was reached resulting in a total of 22 participants. During the recruitment process, it became apparent that other key government Departments (Education and Community and Safety) had limited involvement in the formulation processes, and so respondents from these Departments were not pursued for interviews. This presented a limitation to the study as respondents were largely drawn from the health sector. Follow up interviews were also conducted with two of the key informants from the FTD executive committee a year after initial interviews, to explore if any changes had occurred.

Interviews were undertaken by the first author, guided by a semi-structured interview guide. Respondents were asked about the FTD agenda setting processes, the goals and interventions of the FTD, actor roles and relationships, collaborative processes and contextual factors influencing policy processes. Nineteen face to face and three skype interviews were conducted with informed consent provided by all participants prior to their interviews. Interviews were recorded and transcribed verbatim and lasted between 30 minutes and one hour.

Interview transcripts, outputs of document reviews and field notes from observations were imported into Atlas.ti software. This initial step of data analysis involved generating a timeline mapping the key events associated with the FTD between 2015 and 2019. Further analysis organised data into codes through abduction.^42^ After initial coding a, further interpretation sought to elicit themes through a thematic approach^39^ that explained how key events of the FTD unfolded and why. Our analytical strategy was therefore both inductive based on what emerged from the data and deductive influenced by the Ideas, Interests, Institutions (3Is) framework^43^ to understand the adoption and early implementation experiences of the FTD.

### Ideas, Interests and Institutions Framework

The 3Is framework, draws from a range of theoretical perspectives and identifies the interaction of ideas, interests and institutions as crucial in shaping policy experiences.^43-45^ Ideas refer to how policy problems and solutions are framed and the ability of actors to identify with common goals.^43,45^ Interests encompass the motivations of various actors and the relative sources of power they draw on to influence outcomes.^45^

The concept of institutions has been defined and used in a number of ways.^44,46,47^ Here we draw on the definition of institutions applied to the governance of multisector action.^45^ We refer to institutions as formal laws and the bureaucratic arrangements that govern relationships between different sectoral entities and consider how they, as well as organisational capacity, can affect intersectoral action.^45^ This framework was selected for this study as the three variables assisted in revealing the influence of these critical factors in the FTD experience, especially during adoption and implementation.

## Results

### An Overview of the FTD Policy Process

The FTD initiative reached a peak of attention from political and bureaucratic actors in the Western Cape province in 2016, when it was formally launched by the provincial ministers from the Departments of Health, Social Development and Education. It is worth noting that the FTD was solely a provincial initiative, driven by the provincial health department, and without national budgetary support. Within the bureaucracy, it was agreed that the FTD mandate would be housed under Health Programmes Directorate in the Department of Health, steered by an FTD executive committee (Table 1) responsible for organising the adoption and implementation processes. The FTD was also discussed in the annual plans of both the Departments of Health and Social Development and was included within the multisectoral Provincial Strategic Plan (PSP) (2015-2019),^48^ highlighting its acceptance as a provincial priority.

Policy documents from the health sector state that the intended goal of the FTD in 2016 was “*improving the outcomes of children in terms of health interventions, communication and intersectoral interventions*,”^35^ with budgetary support from the Provincial health department provided for a communication campaign. This goal changed in later years, to include the Survive, Thrive and Transform framework,^30^ as shown in the situational analysis, a key document that proposed an intervention framework to address the FTD.^49^ The attention to the FTD, however, was short-lived, with efforts at cross-sectoral programming declining rapidly from 2018.

In the sections below, we first provide an overview of the agenda setting process of the FTD, and then, using the 3Is framework, we analyse the main contributing factors that led to the thinning of the intersectoral goal. The key events discussed are summarised in Figure.

**Figure:**
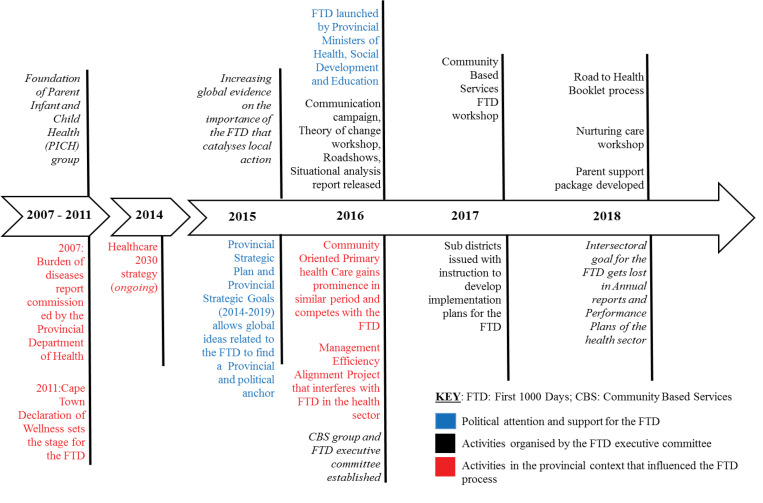
An Overview of the FTD Process With Contextual Processes That Shape the FTD Development and Key Moments Largely Initiated by the FTD Executive Committee. Abbreviation: FTD, first thousand days.

### 1. An Overview of the Agenda Setting Process of the FTD

In the case of the FTD, the multitude of socioeconomic problems and “social ills” in the Western Cape Province had been a core concern of policy-makers for a number of years, such as the high rates of alcohol abuse and related violence which impacts children and families.^38,48^ The need to address safety for children and families had therefore been recognised as a core provincial challenge, although the recognition of the FTD as a key period requiring action was also prompted by the late presentation of patients:

*“So what happened was Red Cross Hospital [tertiary children’s hospital] was saying that they see a lot of children from the ages of three upwards that have conditions that could have been prevented at an earlier age and then they arrive there at three, there is not much…you can do and resources are limited. So we were...trying to do something earlier and then when the FTD came out, it gave us permission to...go aggressively and continue”* (Participant 5, health sector).

Due to the existing local challenges within ECD, global literature showing the impact of the FTD on adult health outcomes, particularly the evidence on brain development in this period,^32^ hit fertile ground in the provincial context and catalysed action towards addressing the FTD. The Survive, Thrive and Transform and the Nurturing Care framework^29,30^ provided policy interventions that could be used to address the FTD. The launch of the “critical 1001 days” in Edinburgh, the global centre of research on child development,^50^ and the SDGs,^24^ were global agendas that were viewed as key:

*“There was an accumulation of evidence over time and a building up of conversations...around the significance of a particular window in terms of long term outcomes. I think the shift in the orientation of the SDGs...and the advent of neuroscience showing...hard traditional scientific evidence which is a lot more digestible and attractive to...practitioners, policy-makers. That kind of evidence reached a critical mass”*(Participant 4, academic).

These global factors,thus, contributed to opening the windows of opportunity for the FTD as they offered it as the suitable vehicle to address ECD problems;and the accompanying frameworks meant that there was a range of various solutions available to address the FTD.

The agenda setting process for the FTD was further enabled by the political atmosphere within the province, that was favourable to the emerging global narratives surrounding the FTD.^51^ The transition to the PSP at the beginning of 2015 offered a new political mood, which centred the value of collaboration and proposed that the security and safety of children and families should be addressed. This allowed the FTD to find a political space or a ‘*home in government’* (Participant 10) implying the FTD and its accompanying solutions made sense within the provincial atmosphere. This was strengthened by the appointment of the provincial health minister in 2015 who was known to be passionate about child health.

Furthermore, participants agreed that “*the soil was tilled and the seeds had been planted* ” (Participant 14) well before 2016, in processes that paved the way for the FTD. The 2007 Burden of Diseases report, signalled a recognition of the impact of SDHs and the importance of focusing on prevention.^32^ The Wellness Summit in 2011 specifically proposed a focus on intersectoral action to address ill health and prioritised attention to child wellness, ensuring early childhood nutrition and creating safe environments as a key requirement of development.^50^ These two processes provided the impetus to begin engaging with concepts of child wellness and triggered the formation of policy communities like PICH.

Child health researchers and clinicians, acting as policy entrepreneurs, used the available intervention frameworks to lobby for attention to the FTD. For example, they created the initial awareness and focus on the FTD, by drawing on their engagement with international literature and global platforms such as the Nurturing Care Framework forum. The PICH group, a policy community (Table 1) was involved in advocacy activities for maternal and child health several years before the FTD initiative. The PICH group is also credited as one of the early communities that drew on the increasing global literature on nutrition and began engaging with the FTD concept through meetings and workshops amongst NGOs, academics and staff from the health department, before 2016. Many perceived that the PICH group continued to have a large influence on the adoption and implementation processes of the FTD and was included in a number of FTD policy documents as a supporting group^52^:

*“The ongoing PICH meetings were also constantly shaping the direction of the FTD initiative, driven by who was showing up”* (Participant 13, NGO).

The ability of certain actors from policy communities such as PICH, to navigate key provincial health spaces was favourable to advancing the FTD agenda, as was the strategic position of actors who supported the FTD idea – such as the Chief Director of Health Programmes who engaged with the top provincial management and the provincial Health Minister, who was a child health advocate.

### 2. Policy Thinning and a Loss of the Intersectoral Goal of the FTD

Child health experts continued to drive a number of other processes after the political launch of FTD, such as a workshop to develop a theory of change for the initiative, a provincial research day on the FTD and a communication campaign, aimed to provide the initiative with an identity and secure FTD onto the provincial government’s agenda. The theory of change workshop was important as it engaged with stakeholders across organisations to develop a plan of action for the FTD, resulting in a complex map of possible interventions. Further events organised by the FTD Executive Committee such as the CBS and Nurturing Care workshops also sought to create awareness and promote a deeper understanding of the FTD concept among various stakeholders.

Despite attempts by the FTD executive committee to continue FTD activities through 2017 and 2018, the loss of the intersectoral focus and goal of the FTD was evident. This is indicated in Table 2, which shows how the FTD focus narrowed over time to activities within the health sector. A previous analysis of policy documents in all relevant sectors for the FTD also revealed limited attention to the initiative beyond the health sector.^51^

**Table 2:** FTD Commitment in the Provincial Department of Health Annual Reports and Annual Performance Plans Between 2015 to 2018

**Provincial Department of Health Documents**	**2015 - 2016**	** 2016 - 2017**	**2017 - 2018**	**2018-2019**
**Annual reports**	“The initiative aims to improve outcomes for children in terms of nutrition, health, education, care/support and parenting, and protection and safety.” “Health specific interventions, intersectoral interventions and effective communication... Whole society approach.”	“Project management plans have been aligned with the Survive, Thrive, Transform framework. **Survive:** 1. Health systems interventions addressing avoidable causes of deaths, 2. Monitoring, evaluation and response system across the care continuum. **Thrive:** Develop a service design framework, wellness maps and Package of Care for the 1st 1000 days. **Transform:** 1. Communication and engagement strategy, 2. Identify and support at risk households in the 4 prioritised geographic areas with inter-sectoral support, via Provincial Strategic Goal 3.”	“The First 1000 Days programme managed by the Department aims to improve performance on maternal and child health indicators.”	“The First 1000 Days programme managed by the Department aims to improve performance on maternal and child health indicators.”
**Annual performance Plans**	“Parenting Programme (first 1000 days), a focused programme on tracking every pregnant women (100000 by year 5) from antenatal care – delivery – post natal care.”	“Designing and implementing a campaign that raises awareness and facilitates action at the community and service provision levels concerning the first 1000 days of a child’s life …Key messages with related actions by parents or main carers and service providers will be determined, using a transversal and multi sectoral approach. The campaign will also promote the important role of men as caring, engaged fathers, supportive partners and carers.”	“Some of the key activities would be: Well baby and child care, quality and links, Development of First 1000 Days initiative social media campaign with information and links for referrals. - Review standard minimum content for antenatal care education that includes addressing issues of substance abuse - Expansion of First 1000 day dashboard (WCG) departments through review process. - Explore and identify areas for research and innovation (Catch and Match & Social Impact Bond). - Intersectoral engagement to encourage departments to promote breastfeeding.”	*No mention of the FTD in the report.*

Abbreviations: FTD, first thousand days; WCG, Western Cape Government.

Policy documents from the health sector mention a health-specific goal for the FTD in 2018 (Table 2) although it was unclear whether health-specific goals presented in policy documents represented the FTD initiative or were ongoing activities of the health sector. Respondent views on the loss of FTD momentum and a limited intersectoral focus are captured in Box 1.

**Box 1. Expressions of the Limited Intersectoral Focus of the FTD**

**Quotes expressing loss of momentum**

*“It has fizzled out….it was an expansive idea, it was meant to be an intersectoral project but there was too little concrete to keep it going. And maybe the intersectoral collaboration killed it, or maybe that’s not fair, there are just so many other confounding things with this case …So the energy that was in FTD is quickly absorbed towards these other concepts of through this design, Management Efficiency Alignment Project restructuring, community oriented primary care”* (Participant 20, health sector).

*“I’m not sure if I saw it translated to kind of programmatic goals so it was kind of quite theoretical still”* (Participant 13, NGO).

*“I’m still hopeful because I think we should invest in the first 1000 days. That’s my criticism of that there isn’t any funding dedicated for this initiative. So we pay somewhat lip service to it by saying we doing something but we [are] actually doing business as usual….in the beginning we had a lot of attention by political ministers…but even those opportunities have now seem to become few and far between. So that’s why I said there’s that little bit of a loss of momentum you know”* (Participant 7, health sector).

*“Maybe I must be blunt and say that the FTD, even though it’s there on paper, is not a priority in the form that was envisaged in terms of... Survive Thrive and Transform. I don’t know if some of that stuff is doable. Let me be honest and say even though we put...in FTD as a priority and we call it a priority, essentially what we’re talking about is maternal services and neonatal services, and then we throw a bit of immunisation in to spice it up a little bit...we are giving attention to that, focused attention to that period, but are we ... fully implementing the recommendations of the task team? The answer is probably, no”* (Participant 19, health sector).

*“We had political buy-in at the very top level. So, Ministers were very happy to be part of the FTD initiative. It had its own branding, it started to be advertised, and then there’s a huge amount of work that had happened the last three years around the FTD. If I look at it objectively, I think it still hasn’t really caught fire beyond Department of Health and maybe to the certain degree in the Department of Social Development”* (Participant 10, clinician).

Abbreviation: FTD, first thousand days.

In the sections below, we examine the reasons why, despite what appeared to be a successful political agenda setting process, the FTD was never anchored as a mainstream multisector strategy in the province. We describe some of the reasons for this experience of policy thinning in relation to the influence of ideas, institutions and interests with the main points highlighted in Box 2.

**Box 2. Summary of Key Findings Using Ideas, Institutions and Interests Constructs**

Ideas

- Ambiguity surrounding the FTD was despite wide awareness of the initiative
- Actors had different ideas regarding interventions for the FTD
- Contention regarding narrow maternal and child health focus versus a broader intersectoral focus
- FTD prioritised in two policy spaces (health sector and intersectoral PSP) which contributed to different ideas regarding interventions

**Institutions**

*Constraints Peculiar to the intersectoral process:*

- Limited consideration of implementation realities during agenda setting:
- Intersectoral activities perceived by implementation players as being unreasonable and outside the boundaries of the health sector especially when the health sector still needed to address its core mandate of ensuring adequate maternal and child health services
- FTD prioritised as a vertical initiative within intersectoral planning spaces

** Broader constraints:**

- Overwhelmed facilities due to increasing patient numbers, limited effective referral systems between sectors and organisations
- Lack of capacity of health staff to engage intersectorally
- Competing policy ideas that were perceived as more feasible such as the Community Oriented Primary Healthcare approach
- The ongoing Management Efficiency Alignment Project that disrupted information and reporting lines in the health sector

** Interests**

- The pressure to address various vertical initiatives such as the FTD overwhelmed implementation actors
- The loss of key champions involved in earlier FTD processes limited the ability of policy entrepreneurs to sustain attention to FTD
- Different interests between policy advocates for the FTD and implementation actors resulted in frustration for both groups of actors

Abbreviations: FTD, first thousand days; PSP, Provincial Strategic Plan.

#### 2.1. Lack of Clarity and Varied Ideas Surrounding the FTD

*“The objectives...and the strategies have been vague and unarticulated and ill defined. So this is not all bad because it allowed for...iterative processes of trial and error. But I have felt...concerned about the wishy-washiness of this initiative. The fact that a lot of people don’t know whether it’s a campaign,...an initiative,...a programme, a lot of people don’t know what it is”*(Participant 4, academic).

The statement above reflects the lack of clarity surrounding the concept of the FTD and difficulty of ascertaining the main objectives of initiative. This was attributed to the fact that the FTD represented a time period in the life course as opposed to a specific programme or policy. Key processes such as the theory of change workshop that were meant to identify specific interventions instead resulted in complex maps that failed to clarify the main activities needed to achieve the FTD goal.

As a result, actors had different ideas regarding policy solutions. The varied policy solutions presented in policy documents revealed three main frames of the problem for the FTD: a biomedical focus, influenced by the health sector; a nurturing care focus linked to ECD; and a socio-economic goal focus reflected by the PSP.^48,51^

The early conceptualisation of the FTD in the two different policy spaces of the provincial government and health department also contributed to the varied meanings of the FTD. Within the PSP the aim was to focus on communication, health interventions and intersectoral interventions.^48^ The PSP provided an intersectoral platform where multiple provincial sectors were expected to engage with the notion of addressing wellness through intersectoral projects meant to be implemented with other sectors. At the same time the FTD was also identified as one of the service priorities of the Department of Health to improve maternal and child health outcomes, by adopting the global Survive, Thrive, Transform framework.^30,49^

This dual conceptualisation of the FTD across policy spaces, described as *“confusedly conceptualised”* (Participant 17), led to differences of views among stakeholders about whether the FTD should primarily focus on maternal and child health aspects as opposed to an intersectoral focus. Most participants felt that intersectoral processes belonged within the PSP space as opposed to within the health sector.

#### 2.2 Institutional Constraints That Shaped the FTD Process

In this section, we distinguish between factors that specifically affected the intersectoral FTD initiative, and those affecting the implementation of other policies led by the health sector.

Constraints Peculiar to Intersectoral Processes

Intersectoral ideas relating to the FTD were viewed as too ambitious for the health sector to undertake due to the limited consideration of complex implementation contexts during agenda setting processes. Moreover, intersectoral activities were also viewed to be intangible and outside the boundaries of the health sector, making them difficult to engage with for actors at service delivery level. Some felt that intersectoral action should not be a key focus when the health sector even struggled to ensure child survival that was its core mandate:

*“I don’t recall ever agreeing that we’ve got past the survive [child survival] part,...I don’t know, we’re talking about transformation [intersectoral action] and there’s still children dying...we need to get the basics right. And I don’t think the basics are there”* (Participant 19, health sector).

The historical pattern of prioritising vertical projects managed by specific sectors within planning spaces that were meant to be intersectoral, such as within the PSP, was also felt to have impacted the FTD. The FTD was prioritised as one of the vertical initiatives along with six other similar initiatives spread across three government sectors, setting the precedent for how it unfolded as a vertical initiative within the health sector. This was worsened by how ECD services were fragmented across various sectors, and within NGOs contracted by individual sectors, resulting in the duplication of services.

*“It [The FTD] was landed in the...supposedly inter-sectoral space as a vertical project, alongside vertical projects of other departments. So that was the conceptualisation of the FTD as a parallel project within ostensibly an inter-sectoral space”*(Participant 17, health sector).

Broader Institutional Constraints

Systemic support for the FTD was hindered by service-delivery contexts shaped by extensive social disparities, high patient numbers, and ineffective referral systems. Similarly, some respondents felt the FTD focus on parental support and empathetic care was undermined by poor provider skills and the traditional focus on record keeping over patient engagement. These systemic challenges, combined with the lack of clarity surrounding the concept of the FTD, led senior managers to resist the FTD:

*“We’re going to buffer the services [health workers] from this [the FTD]. Because they can quite easily focus a hundred percent of their time on this and then everything else collapses... So the concern is that inappropriate focus without good planning and ...prioritisation will lead to us providing a service which is not commensurate with the needs of the population”* (Participant 19, health sector).

The FTD also appears to have lost attention as other policy ideas within the health system arena that were regarded as more tangible, such as the community oriented primary care (COPC) approach, gained prominence during the same period. The COPC approach is built on the provision of primary healthcare services in co-ordinated geographical locations or communities.^53^

*“COPC is robust, it’s been around for a long time. It doesn’t really need anyone to fight for it. It just needs an aha moment which has now happened and it will emerge naturally from the system. So I think the difference is, one is an idea*[the FTD]*, and the other is far more tangible”*(Participant 20, health sector).

At the same time, a wider organisational restructuring process, the Management Efficiency Alignment Project,^54^ disrupted information and reporting lines in ways that were felt to have undermined the focus on the FTD:

*“It’s a bit loose fitting at the moment in my opinion. …Most of us are at least loose fitting in the Department. You are not sure where you fit in the future structure. People feel that it’s that floatingness…I’m not sure where it’s going to end up…so must I take it [The FTD] forward is it worthwhile? Who is going to support this? Is it going get the attention that it requires for me to put that effort?”* (Participant 7, health sector).

#### 2.3 Interests, Actors’ Tensions and Resulting Frustrations

The two key groups of actors who were influential in the FTD policy process were policy entrepreneurs, involved in interest groups and policy communities such as PICH, and actors at the service-delivery responsible for the implementation of programs within the health system. The FTD process revealed underlying tensions between them at both provincial and district levels.

At the provincial level, contestation emerged during decisions-making processes regarding interventions, such as during the design of community health worker (CHW) packages for the FTD. The demands of the FTD interest groups and policy communities regarding the set of interventions that CHWs should perform conflicted with the opinion of service managers regarding what was feasible for CHWs. Service managers felt that including a large set of FTD interventions would negatively influence the provision of other services, while policy entrepreneurs pushed for a focus on various specific interventions, depending on their interests.

At the district and sub district level, implementation actors had to navigate between multiple demands from top managerial structures such as the implementation of other priority focus areas like immunisation programs, and from interest groups advocating for various vertical initiatives. One of the respondents expressed the pressure faced in this manner:

*“There is this group for the FTD, there are groups like this for mental health, there are groups like this for chronic disease management… All those groups come and all those groups want a piece of you, so if we are not doing enough for children.., then they criticize but not understanding that [the] same resources has to provide [for] other services as well”* (Participant 5, health sector).

Participants also identified a range of missing actors who many felt could have had a positive impact on the realisation of the FTD goals. These included actors from the Departments of Social Development and Education as well as representatives of human resources and finance departments who would be necessary to support the goals of the FTD. Although the FTD was noted as a priority for Social Development sector in policy documents from 2016 to 2018,^55–57^ there was limited attendance of actors from the department in forums such as PICH.

The ability of the FTD policy entrepreneurs to lobby within senior management structures of the health sector was also negatively impacted by the resignation of a key senior champion of initiative:

*“My sense is that there’s particular people that are passionate about specific initiatives and that person had a passion to see this as a key priority competing with all the other multiple priorities, but because that person’s voice is no longer in top management meetings...There is a little bit [of] a loss for this initiative”*(Participant 7, health sector).

The gradual loss of other actors who had been involved in the initial formulation activities, only worsened the situation.

As a result, many respondents felt that there was limited ownership of the initiative by the actors responsible for service delivery, and judged that they largely viewed the FTD as an externally driven agenda of the policy entrepreneurs. Senior management structures then felt they had to *“protect”* service providers against the FTD, with one participant wondering *“what planet”* policy advocates for the FTD were in and wanted to *“reign them in”*(Participant 19). Implementation actors were also frustrated by the expectation of having to implement an intersectoral initiative that had unclear interventions and their inability to express their discontent of the process:

*“A lot of feedback that you would get on this topic is somewhat…it’s about mothers and babies… and you can’t express your frustration on that. You can’t say that you are skewing the system and pulling resources from other places. I think there was a lot of quiet resentment about this topic, because you’re not allowed to express your frustration with the modus operandi”* (Participant 20, health sector).

On the other hand, for policy entrepreneurs who had been involved in lobbying for attention to the FTD for a long period of time, the loss of momentum to the FTD was a source of frustration due to the lack of institutional support and fading political support.

Interaction of Ideas, Interests and Institutions

In summary, the ideas surrounding the FTD initiative arose from the interests of policy entrepreneurs and the FTD was prioritised as a vertical (health-based) initiative due to historical patterns of priority setting within sectors. The proliferation of vertical initiatives such as FTD created resistance among implementation actors (interests) who had to navigate multiple demands and the systemic challenges of the health sector (institutions). Implementation actors, who largely had decision-making power over implementation activities, therefore, behaved in ways that resisted the intersectoral goal of the FTD (interests) and instead focused on health-based mandates which seemed to fit with their perceived idea of what the health sector could manage (institutions). The designation of the FTD mandate to the health sector (institutions) and limited engagement with other sectors further isolated the FTD as another health intervention (ideas) and the FTD appeared to loose relevance even within the health sector, given other policy ideas (institutions). The interaction of unclear ideas and vertical initiatives, institutional constraints and challenges that affected the process and divergent interests between policy entrepreneurs and system actors therefore resulted in the loss of the intersectoral mandate of the FTD.

## Discussion

This paper set out to explore what hinders the implementation of intersectoral approaches despite successful political agenda setting. Applying the 3Is framework, we show how, in this experience, child health researchers and clinicians, as policy entrepreneurs, maximised windows of opportunity favoured by global evidence and local contexts. However, the prioritisation of the FTD as a vertical initiative, along with limited cross-sector engagement and the lack of consideration of implementation realities, set the precedent for the loss of intersectoral action in later years. The ambiguity surrounding interventions for the FTD, institutional barriers and the resistance to intersectoral goals by implementation actors contributed to the narrowing of action. Our discussion focuses on these key themes from the Western Cape province FTD experience and draws attention to what is needed to advance efforts towards the realisation of intersectoral approaches.

A number of policy studies have outlined agenda setting as a crucial stage in policy processes, and identified political attention as a key ingredient in facilitating attention to policy issues.^9,10,22^ The FTD experience prompts the reflection that initial political attention without bureaucratic commitment to action at multiple levels may not sustain action over time. Similar to the FTD initiative, previous experiences have shown how despite prioritisation, policy intentions can become arbitrary with inactive planning committees,^22^ can fluctuate on and off the agenda^11^ or collapse after a few years.^54^

These experiences draw attention to the relevance of agenda setting in setting the stage for subsequent policy processes, particularly for the negotiation of favourable policy options. In the case of intersectoral approaches, the prioritisation of policy issues as vertical projects, because that was the way things were done traditionally, emerged as a key hindrance to ensuring collaboration during FTD implementation, especially as cross-sector partners were not involved at agenda setting.In other experiences,^9,23^ the transition between a vertical project to an institutionalised intersectoral approach became a major constraint to implementation. The challenge is greater when the health sector adopts the responsibility for the initiative, as this makes it difficult for other sectors to get involved when the initiative is viewed as a health program.

Due to its regular contact with children from birth, the health sector is expected to play a crucial stewardship role in the FTD.^25^ However, our analysis reveals how the institutional constraints of the health sector influence the interests and actions of actors within this FTD experience. Given also the ambiguity of the FTD, implementation actors perceived interventions based on health sector mandates as most feasible to implement and so supported ideas that fitted with the health sector (preference for health interventions over multisector efforts). Similarly, a biomedical perspective of health has been shown to be dominant within health systems, even for issues that require broader societal focus.^12,58^ For example, resources are used in ways that favour biomedical and individualised interventions to address SDH policy issues despite acknowledgement in the discourse of health policies of the need for intersectoral approaches.^12,58^ The prioritisation of biomedical care therefore impacts the ability of health sectors to engage in intersectoral action.^15^

The tension between focusing on health-based interventions versus multisectoral efforts has also emerged elsewhere.^10^ Decisions regarding which interventions will be predominant depends on the sector that controls the agenda and defines the policy problem as well as perceptions regarding the feasibility of interventions.^10^ Debating broad versus narrow interventions can be an advantage if existing forums allow discussions and for the negotiations of options. However, in the absence of effective institutional mechanisms that allow the deliberation of available options for intersectoral action, decisions are often made in ways that suit the dominant organisation,^59^ which in this case was the health sector.

The role of the health sector in intersectoral approaches is important to consider particularly as the health sector often carries the responsibility for addressing a number of wicked problems^15^ such as the FTD. Experiences from this study confirm that assuming that the health sector should bear the responsibility of initiating, managing and sustaining intersectoral action on its own is unreasonable.^15^ Existing fragmentation along program lines and budgets within health systems does not create conducive institutional spaces for cross-sector engagements.^15^ It is therefore worth considering ways that the health sector can sufficiently engage with other sectors. Some have proposed that the distinction between which sector owns particular policy issues can be the first step in ensuring more meaningful cross-sector engagement. De Leeuw for example, proposes the identification of societal issues owned solely by the health sector, those that can be initiated by the health sector but co-owned by others and those owned by other sectors with possible health sector input.^15^ However, the negotiation of these arrangements requires the consideration of how to establish supportive cross-sector spaces of engagement.

The link between institutions and actors’ interests is valuable to explore in intersectoral approaches, where multiple organisations with different interests need to collaborate.^23^ A number of policy experiences recommend the need for cross-sector structures that promote dialogue and the negotiation of different views. These have been referred to as interdisciplinary committees, working groups^60^ or policy networks.^61,62^ The establishment of cross-sector groups can enable intersectoral collaboration by providing the space to negotiate different perspectives between various actors within the government and non-state actors.^60^ Baum et al refer to ‘supportive bureaucratic policy networks’ that include senior and mid-level staff across sectors as a powerful way to facilitate cross-sector engagements and bring about action on the SDHs.^23^

Apart from being valuable in providing deliberation spaces for actors across organisations, networks can contribute to changes in norms, preferences, interests through the continuous engagement and dialogue amongst actors.^61,62^ The collaborative governance literature also presents collaboration as an iterative process that requires careful planning and posits that collaboration may be more effective if relationship- building is prioritised amongst actors.^63,64^ Similarly, others acknowledge that investing the time to build and sustain relationships can enable the development of common goals that embrace collective interests.^14,23^

Collaborative networks can also be valuable spaces for implementation actors at service delivery level. This FTD process reveals the risks of implementing actors having limited spaces and opportunities to voice opinions on policy processes. This can lead to front-line actors interpreting and adapting policy in ways that can result in unexpected outcomes.^65^ Networks that include both bureaucratic actors and actors outside the government can be advantageous for policy entrepreneurs in cases such as FTD, as it avoids external actors being seen as outsiders and having limited decision-making power on proposed interventions.^66^ Spaces for negotiating different understandings with implementation actors from different sectors can take forms that vary depending on the need and the groups of actors involved.^66^ Achieving the level of co-ordination required for collaboration however, requires clear definitions of the policy problem in order to establish ownership amongst different partners.^63,67,68^ A vague construction of the problem and solution, as shown within this FTD experience, makes it difficult to establish the necessary partnerships and build consensus around shared goals that slows policy momentum. Establishing clear and common goals is particularly important at early stages of policy-making as it sets the stage for subsequent implementation processes. Implementation processes are complex and messy and require clear objectives and adequate resources.^6,43^ Implementation processes also engage a wide range of actors at the service-delivery level which makes it difficult to operationalise unclear policy objectives. Failing to address unclear objectives can result in policy stasis and the failure of collaborative efforts can create distrust and suspicion among stakeholders which may affect future collaborative endeavours.^58,63^

Building on this analysis of the Western Cape FTD experience additional research could usefully seek to understand the perspectives of other sectoral players, and their respective institutional barriers and opportunities in similar initiatives. The advent of the SDGs offers the platform to advance intersectoral collaboration through the interlinkages between goals; however, several policy experiences^9,22,23^ suggest the need to examine challenges that can threaten the advancement of the SDGs. A particular risk is the adoption of vertical approaches for each goal, especially as the allocation of resources often falls across several sectors. Efforts to address the SDGs therefore need to consider the value of building common understandings between relevant sectors, ensure that actors have appropriate policy spaces that allow for policy dialogue and the negotiation of different perspectives, and pay attention to the principles of effective collaborative governance^63,64^ that allow for meaningful cross-sectoral engagements.

## Conclusion

This research illustrates what factors influence policy adoption and early processes of implementation for intersectoral approaches. The analysis has been helpful in drawing out the conditions which should be considered for the effective implementation of intersectoral action, including for the FTD initiative as a continuing priority of the Western Cape province. Firstly, lessons from this study emphasise the importance of including cross-sector players at agenda setting and the value of engaging with implementing actors to ensure ownership of initiatives. Secondly, we highlight that the vertical prioritisation of intersectoral approaches can hinder attempts to institutionalise collaboration at later stages of the policy process. We draw attention to the need for system-wide commitment beyond political attention, especially the allocation of financial and human resources to ensure the realisation of intersectoral goals.

Finally, we propose that the negotiation of the interests of various sectors and of government and non-government actors can be promoted through sustained engagements amongst stakeholders in networks that prioritise relationship building and trust. Collaborative processes founded on trust and effective communication allow the emergence of common goals that embrace collective interests. Networks that include relevant bureaucratic actors can foster shared decisions and may alleviate tensions regarding interventions.

## Acknowledgements

We acknowledge the support of key informants involved in the FTD initiative who shared their experiences and key documents that shaped the analysis of this case. This paper has received support from the Health Policy Analysis Fellowship programme of the Alliance for Health Policy and Systems Research, Switzerland.’ We would also like to thank the Health Policy Analysis fellows for support and feedback on the paper. As well as Professor Lucy Gilson and Dr. Zubin Schroff for their insights on later versions of the paper and for reviewing the manuscript prior to submission.

## Ethical issues

All data collection activities received prior ethics approval from the University of the Western Cape Biomedical Health Committee (BM17/10/9) and the Provincial Department of Health (WC_201712_026).

## Competing interests

Authors declare that they have no competing interests.

## Authors’ contributions

Overall design of the study: IO, UL, HS. Data collection activities: IO. Analysis, conceptualisation of the manuscript: IO, UL, HS. Writing the original manuscript draft: IO. Review and editing of subsequent manuscript drafts: IO, UL, HS. All authors read the manuscript and approved the final version of the manuscript.

## Authors’ affiliations

^1^Department of Community and Health Sciences, School of Public Health, University of the Western Cape, Cape Town, South Africa. ^2^UWC/SAMRC Health Services to Systems Research Unit, School of Public Health, University of the Western Cape, Cape Town, South Africa.

## Funding

This work is based on the research supported by the South African Research Chairs Initiative of the Department of Science and Technology and National Research Foundation of South Africa (grant no. 98918). The authors would also like to acknowledge funding from the South African Medical Research Council and the Belgian Development Cooperation, through the Institute of Tropical Medicine, Antwerp. Any opinion, finding and conclusion or recommendation expressed in this material is that of the authors and not the funders.

## Key Messages

**Implications for policy makers**

This study prompts policy-makers to consider the following factors when developing policies requiring intersectoral approaches:

- High-level political attention to intersectoral action requires system wide commitment at sub national and implementation levels. The ownership of intersectoral mandates by implementation teams at district level is crucial for effective implementation.
- The transition from intersectoral approaches that are adopted as vertical programs to instutionalised approaches to address social determinants of health (SDH) can hinder implementation. Policy-makers therefore need to consider ways to engage with intersectoral partners in the priority setting stage and to sustain these relationships throughout the policy process.
- Negotiation between actors and sectoral interests can benefit from long-term engagement processes that support relationship-building and trust development amongst stakeholders, including through policy networks that include bureaucratic actors across sectors. These engagements can enable navigation towards collective goals by embracing collective interests.
- Institutionalising intersectoral action not only requires existing and supportive policy networks but also the financial and human resources needed to support the creation of an enabling environment and to sustain intersectoral approaches.

**Implications for public**

This study highlights the challenges and opportunities that need to be considered in developing intersectoral approaches aiming to address the social determinants of health (SDH). Considering these factors when developing and implementing initiatives similar to the first thousand days (FTD) can ultimately improve their implementation.
